# Evaluating the effectiveness of transferrin receptor‐1 (*TfR1*) as a magnetic resonance reporter gene

**DOI:** 10.1002/cmmi.1686

**Published:** 2016-02-29

**Authors:** Sofia M. Pereira, Anne Herrmann, Diana Moss, Harish Poptani, Steve R. Williams, Patricia Murray, Arthur Taylor

**Affiliations:** ^1^ Institute of Translational MedicineUniversity of LiverpoolLiverpoolUK; ^2^ Institute of Integrative BiologyUniversity of LiverpoolLiverpoolUK; ^3^ Centre for Imaging Sciences, Oxford RoadUniversity of ManchesterManchesterUK

**Keywords:** MRI, reporter gene, cell tracking, transferrin receptor, ferritin, chick embryo, CHO‐K1

## Abstract

Magnetic resonance (MR) reporter genes have the potential for tracking the biodistribution and fate of cells *in vivo*, thus allowing the safety, efficacy and mechanisms of action of cell‐based therapies to be comprehensively assessed. In this study, we evaluate the effectiveness of the iron importer transferrin receptor‐1 (*TfR1*) as an MR reporter gene in the model cell line CHO‐K1. Overexpression of the *TfR1* transgene led to a reduction in the levels of endogenous *TfR1* mRNA, but to a 60‐fold increase in total TfR1 protein levels. Although the mRNA levels of ferritin heavy chain‐1 (*Fth1*) did not change, Fth1 protein levels increased 13‐fold. The concentration of intracellular iron increased significantly, even when cells were cultured in medium that was not supplemented with iron and the amount of iron in the extracellular environment was thus at physiological levels. However, we found that, by supplementing the cell culture medium with ferric citrate, a comparable degree of iron uptake and MR contrast could be achieved in control cells that did not express the *TfR1* transgene. Sufficient MR contrast to enable the cells to be detected *in vivo* following their administration into the midbrain of chick embryos was obtained irrespective of the reporter gene. We conclude that *TfR1* is not an effective reporter and that, to track the biodistribution of cells with MR imaging in the short term, it is sufficient to simply culture cells in the presence of ferric citrate. Copyright © 2016 The Authors *Contrast Media & Molecular Imaging* Published by John Wiley & Sons Ltd.

## 1 Introduction

Cell‐based therapies have enormous potential to treat conditions that are refractory to other forms of therapy, including various degenerative diseases and cancer 1. The efficacy of cell‐based therapies has already been demonstrated in numerous preclinical studies, but lack of knowledge pertaining to the biodistribution and fate of the cells poses a major safety concern, and is an important issue preventing the translation of these promising therapies to the clinic 2. The imaging modalities that can be used to track the biodistribution and fate of administered cells *in vivo* include bioluminescence, fluorescence, photoacoustic, nuclear and magnetic resonance (MR) imaging 3. Although all of these modalities can be used to image cells in small animals, only nuclear and MR imaging have the penetration depth required to image cells within the internal organs of humans. A drawback of nuclear imaging techniques, however, is that they suffer from poor spatial resolution, and perhaps more importantly require exposure to ionizing radiation, which is particularly problematic for longitudinal studies that necessitate repeated scanning. MR imaging (MRI), on the other hand, which has very high spatial resolution and an excellent safety profile, has already been used to successfully track labelled cells in animal models and in humans 4, 5.

Most MRI‐based cell tracking studies to date have focussed on imaging cells labelled with MR contrast agents, such as superparamagnetic iron oxide nanoparticles (SPIONs) or fluorine‐based compounds (^19^F) 4, 6. While these direct cell labelling approaches can be very good for monitoring the biodistribution of cells in the short term, they are not suitable for long‐term cell tracking, the main reasons being that (i) their concentration in the cell is diluted by 50% with each cell division, which means that after several cell divisions they may become undetectable 7, (ii) they can degrade within the cell, (iii) if the labelled cell dies, the contrast agent can be taken up by host cells, leading to false positive results 8, 9 and (iv) they can only be used for monitoring cellular biodistribution and give no indication of cell phenotype or differentiation status. For these reasons, there has been an increasing interest in exploring the possibility of using MR reporter genes for cell tracking 10. The advantage of reporter genes is that the intensity of their signal is maintained with each cell division, and can rapidly disappear if the cell dies, thereby facilitating the detection of viable cells and avoiding the potential problem of detecting false positives. Furthermore, reporter genes can be used for a more diverse set of applications depending on the promoter used to drive their expression; for instance, constitutive promoters can be used for biodistribution studies, whereas cell‐type‐specific promoters can indicate the cell's differentiation status.

A range of MR reporter genes have been proposed 10, but the majority of the systems used to date are involved in the regulation of iron homeostasis. Overexpression of such reporters facilitates cell tracking by increasing the intracellular concentration of iron to levels that are high enough to generate detectable MR contrast. Mammalian MR reporters include the transferrin receptor, which is required for importing iron into the cell 11, 12, and ferritin, which is required for iron storage 13, 14. To ensure iron homeostasis is maintained, the expression of the transferrin receptor and ferritin genes is tightly regulated at both the transcriptional and translational levels 15. For instance, when intracellular iron concentrations are low, expression levels of the transferrin receptor increase so that iron can be repleted through the import of holo‐transferrin (i.e. iron‐bound transferrin) from the extracellular environment. Concomitantly, levels of ferritin decrease so more iron is released from ferritin iron stores into the labile iron pool (LIP). When intracellular iron stores are high, the reverse events happen; i.e., transferrin receptor expression decreases to reduce levels of imported iron, and ferritin levels increase, so that the excess iron is safely stored within ferritin complexes 16.

We have recently investigated if overexpression of ferritin heavy chain‐1 (*Fth1*) and transferrin receptor‐1 (*TfR*1) in mouse mesenchymal stem cells (mMSCs) affects iron homeostasis and cell phenotype 17. In that study, we reported that, at physiological concentrations of extracellular iron (i.e. in the absence of ferric citrate supplementation), overexpression of *Fth1* reduced proliferation and led to changes in cell phenotype, suggesting that *Fth1* might only be suitable for tracking cells in organs and tissues where iron levels are sufficiently high to maintain the cell's viability. *TfR1* overexpression, on the other hand, was well tolerated by the mMSCs and did not cause any obvious adverse effects on the cell's phenotype. Unexpectedly, however, overexpression of *TfR1* did not increase intracellular iron levels in mMSCs, making it an ineffective MR reporter in this cell type 17. It is possible that the failure of *TfR1* to operate as an MR reporter in mMSCs might have been because the expression levels of the recombinant *TfR1* protein were insufficient. Thus, to further explore the potential of *TfR1* as an MR reporter, in the current study we have used a bicistronic TfR1:GFP lentiviral vector to induce high levels of *TfR1* expression in Chinese hamster ovary (CHO) cells. CHO cells are known for their ability to express high levels of recombinant protein 18, and thus present an ideal test system for evaluating the *TfR1* reporter. Using this CHO reporter line, we have investigated the effect of *TfR1* overexpression on iron homeostasis *in vitro*, and evaluated the effectiveness of *TfR1* as an MR reporter *in vivo* using a 3Rs‐compliant chick embryo model.

## 2 Results

### 2.1 Expression of a bicistronic TfR1:GFP reporter in CHO‐K1 cells

**Figure 1:**
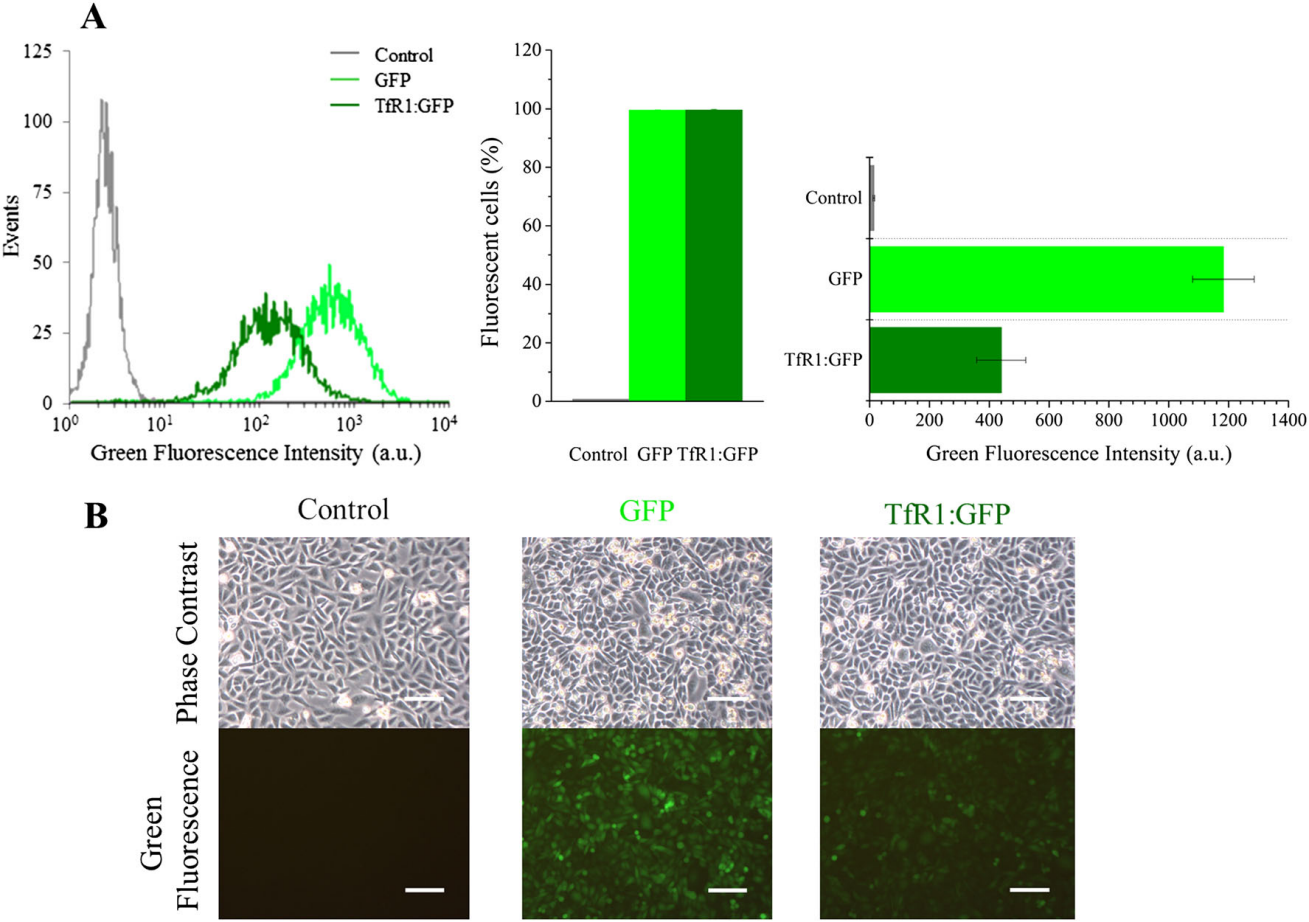
Transduction efficiency of CHO‐K1 is almost 100% with both GFP and TfR1:GFP lentiviral particles. (A) Flow cytometry analyses show successful integration of the transgenes (histogram, fraction of positive cells and the green fluorescence intensity). (B) Phase contrast and fluorescence images of CHO‐K1 cells that were transduced with each of the constructs. Scale bars correspond to 100 μm.

To enable *TfR1* transgene expression to be monitored in CHO‐K1 cells using flow cytometry and fluorescence imaging, mouse *TfR1* was cloned upstream of IRES‐GFP under the control of the constitutive promoter EF‐1α, as previously described 17. The *TfR1* iron response element was removed prior to cloning to ensure efficient translation of the TfR1 protein; this well‐established strategy allows circumvention of the negative feedback loop that prevents the accumulation of TfR1 protein when the concentration of intracellular iron is high 19, 20, 21, 22. Flow cytometry showed that the transduction efficiency with both TfR1:GFP and GFP control lentivirus was almost 100% (Fig. 1A). The fluorescence intensity of the TfR1:GFP transduced cells was slightly lower than that of the cells transduced with GFP alone, which is probably due to the differences in construct size and the fact that GFP is expressed downstream of IRES. Fluorescence microscopy confirmed the results obtained with flow cytometry (Fig. 1B).

### 2.2 Effect of *TfR1* reporter expression on *TfR1* and *Fth1* mRNA and protein levels

**Figure 2:**
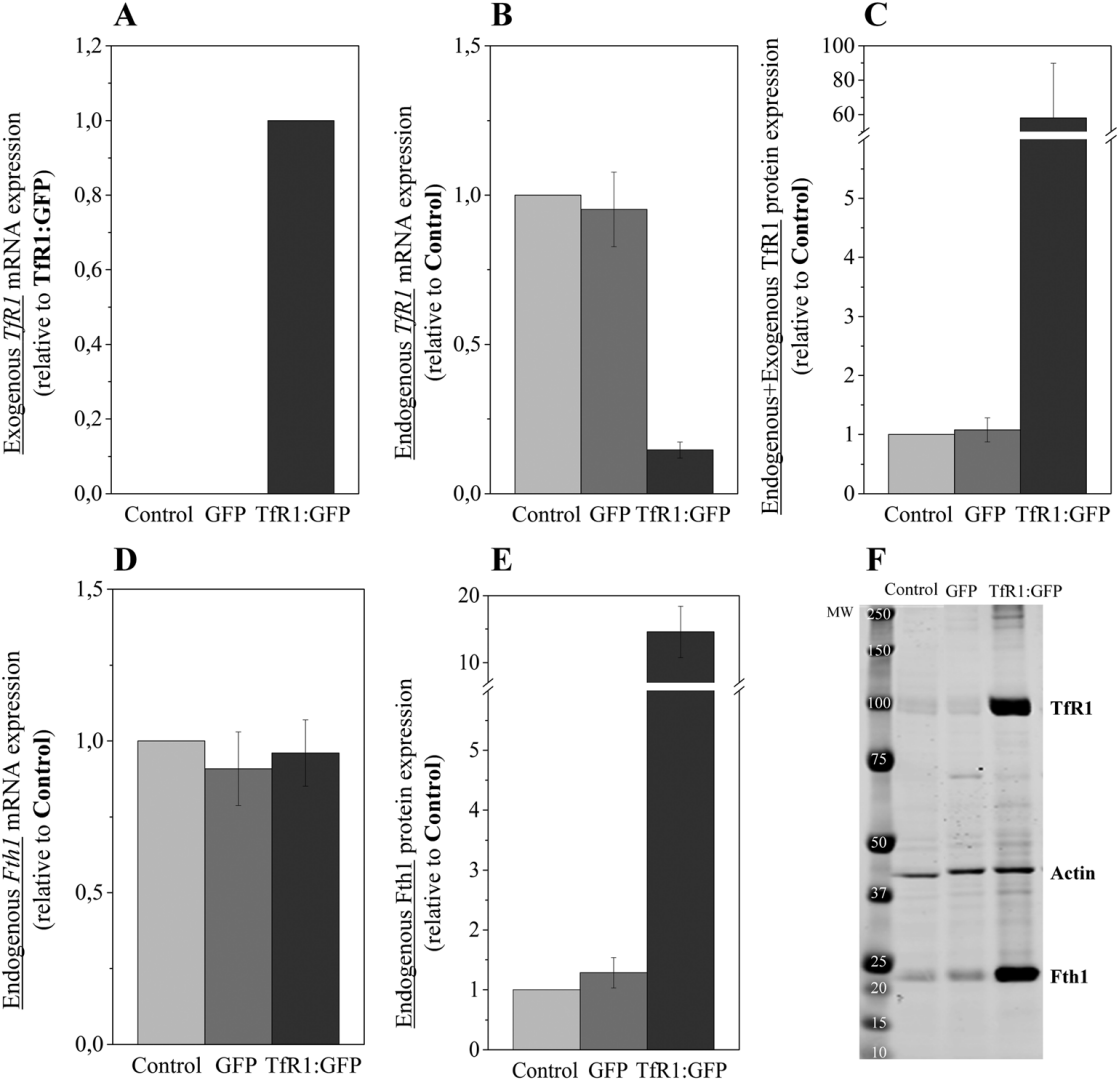
Overexpression of *TfR1* transgene leads to a significant down‐regulation of endogenous *TfR1* mRNA and a dramatic increase in levels of TfR1 and ferritin protein. Relative quantification of (A) exogenous *TfR1* transcripts, (B) endogenous *TfR1* transcripts, (C) total TfR1 protein, (D) *Fth1* transcripts and (E) Fth1 protein. (F) Representative western blot. Error bars represent SEM (*n* = 3).

RT‐qPCR analysis confirmed robust expression of the mouse *TfR1* reporter in CHO‐K1 cells (Fig. 2A). Using specific primers to distinguish between mouse and hamster *TfR1*, RT‐qPCR showed that overexpression of mouse *TfR1* caused a significant down‐regulation of endogenous *TfR1* in the CHO‐K1 cells (Fig. 2B). Nevertheless, despite the reduced levels of endogenous *TfR1* mRNA, overexpression of the mouse transgene resulted in an approximately 60‐fold increase in TfR1 total protein levels (Fig. 2C, F). Although overexpression of *TfR1* did not affect *Fth1* mRNA levels (Fig. 2D), an approximately 13‐fold increase in Fth1 protein levels was observed (Fig. 2E, F).

### 2.3 Effect of *TfR1* reporter expression on intracellular iron content

**Figure 3:**
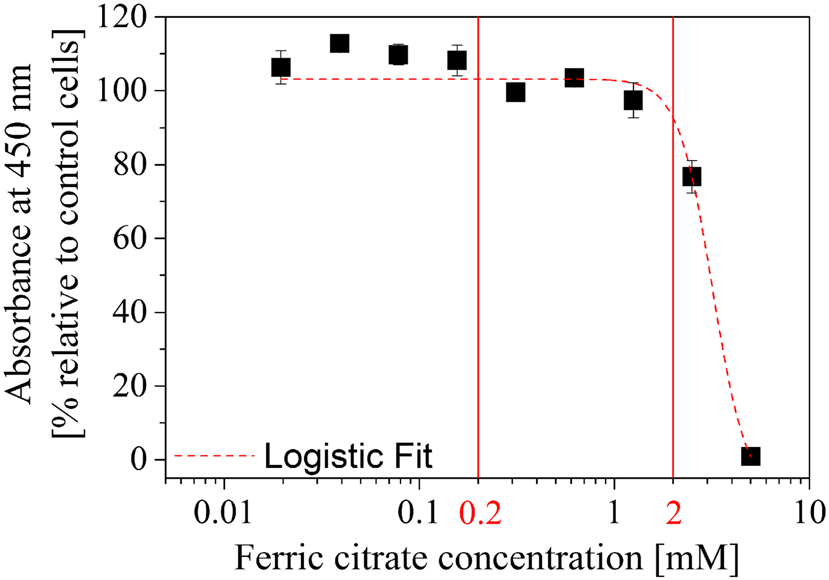
The viability of CHO‐K1 cells after exposure to ferric citrate. Cells were exposed to FC for 24 h and the viability was quantified using a colorimetric assay based on the water‐soluble tetrazolium salt WST‐8. Viability is expressed as the absorbance at 450 nm in relation to control (untreated) cells. Error bars represent the SD from three measurements and the concentrations used as a supplementation for iron loading are shown in red. A maximum supplementation of 2 mM FC, where viability was above 90%, was chosen for all experiments.

**Figure 4:**
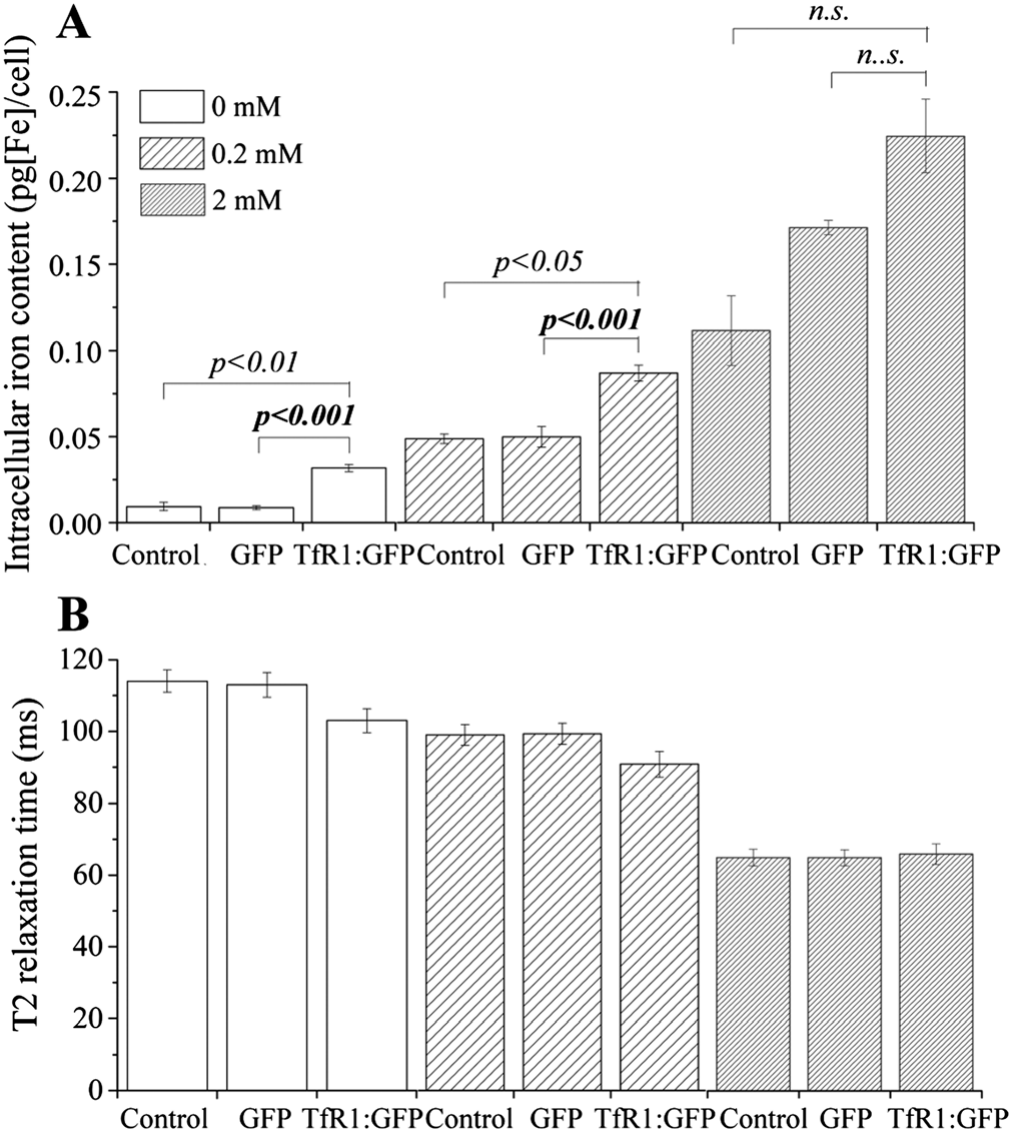
The effect of *TfR1* reporter on the (A) intracellular iron content and (B) *T*_2_ relaxation of CHO‐K1 cells. Control and transduced cells were cultured for 72 h with regular medium or supplemented with ascorbic acid, human holo‐transferrin and ferric citrate at a concentration of 0.2 or 2 mM. Error bars represent the SEM (*n* = 3) for the intracellular iron content and the pixel‐based SD for the *T*_2_ relaxation. The relaxation data (signal versus *TE*) are presented in Fig. S3.

The ability of the *TfR1* reporter to increase the intracellular iron content of CHO‐K1 cells was investigated under conditions where the concentration of extracellular iron was increased by supplementation with 0 mM (control), 0.2 mM or 2 mM ferric citrate (FC), the maximum concentration of FC that the cells tolerated without displaying any toxic effects (Fig. 3). The latter two conditions were additionally supplemented with 50 μM ascorbic acid and 1.28 mM human holo‐transferrin, as previously described 17, which allows maximum intracellular iron accumulation (Fig. S2). The basal medium used here contains only approximately 250 nM of iron. In the absence of FC supplementation, cells displayed an intracellular iron content of about 0.01 pg/cell, which significantly increased to about 0.03 pg/cell when expressing *TfR1* (Fig. 4A). Likewise, in the presence of 0.2 mM FC, the iron content of TfR1^+^ cells was significantly higher than that of controls (0.09 pg/cell compared with 0.05 pg/cell). Although a further increase in the iron content of the TfR1^+^ cells was observed in the presence of 2 mM FC, it was not significantly different from controls (~0.22 pg/cell compared with 0.11 pg/cell) (Fig. 4A). Differences in iron content were reflected in the *T*_2_ relaxation time of the cell pellets, which was reduced with respect to controls for cells expressing the *TfR1* reporter, when cultured in the absence of FC supplementation or in the presence of 0.2 mM FC (Fig. 4B). Of note, although these results show that the *TfR1* reporter can effectively increase the intracellular iron content of CHO‐K1 cells, the observed increase was quite modest, and a much greater increase in iron content and reduction in relaxation time could be achieved by simply supplementing the culture medium with FC; for instance, in the presence of 2 mM FC, the iron content of untransduced CHO‐K1 cells increased from about 0.01 to 0.11 pg/cell (Fig. 4A) and was associated with a substantial reduction in transverse relaxation time (Fig. 4B).

### 2.4 MRI

**Figure 5:**
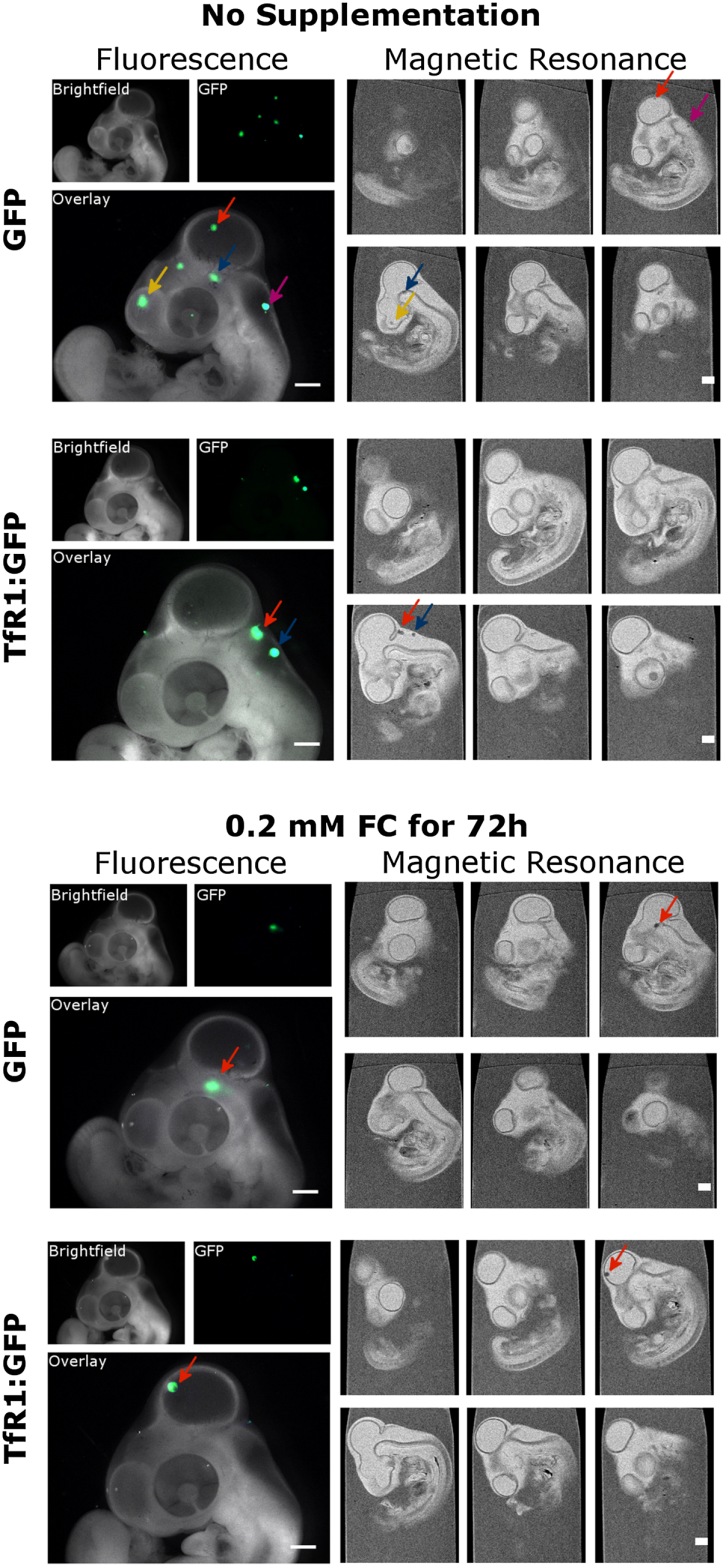
Fluorescence and MR imaging of cells implanted into the brain of a chick embryo. Cells (2 × 10^5^) expressing GFP or TfR1:GFP were implanted into the midbrain of chick embryos at E3. At E5 the embryos were harvested from their eggs, imaged with a fluorescence stereomicroscope and fixed prior to MRI using a *T*_2_‐weighted RARE sequence. Clusters of cells expressing GFP were found in different regions of brain via fluorescence microscopy and are indicated with arrows. These clusters could then be identified as hypointense regions in the MR images. Six sagittal slices through the chick are shown in the right‐hand panel. Scale bars represent 1 mm.

To evaluate the effectiveness of both the *TfR1* reporter and extracellular iron supplementation for facilitating MRI‐based cell tracking, CHO‐K1 cells transduced with the GFP construct (controls) or the bicistronic TfR1:GFP reporter were injected into the midbrain of chick embryos *in ovo* at Embryonic Day 3 (E3). Both cell types were cultured in the presence of 0 mM, 0.2 mM or 2 mM FC for 3 days prior to injection. At E5, embryos were imaged using fluorescence microscopy to identify the location of the transplanted GFP^+^ cells. MRI was then performed to see if overexpression of the *TfR1* reporter and/or FC supplementation enabled these same cells to be visualized with both the fluorescence and MR imaging modalities. As we have previously shown 7, 17 cells injected into a single site at E3 are often found in the form of several clusters at E5 (Fig. 5), which is a consequence of the quick brain development during this period. These conditions offer the chance to monitor the migration of cells and, in combination with fluorescence imaging, which enables the tracking of cell viability, ensure that any MR contrast is not a result of the carryover of iron to a single site or the accumulation of iron from dead cells. In all conditions, it was possible to detect a loss in MR signal at the sites where clusters of cells expressing either GFP or TfR1:GFP were detected via fluorescence imaging (Fig. 5 and Fig S4). Although it might appear surprising that GFP^+^ cells cultured in the absence of FC supplementation could be detected via MR, it is important to note that the *T*_2_ relaxation time of the chick embryo's brain is relatively long. Our own measurements and previous reports 23 suggest relaxation times over 200 ms at this embryonic stage and thus much longer than we have measured for GFP cell pellets cultured in the absence of FC (114 ms, Fig. 4B). The loss of signal for clusters of cells expressing TfR1:GFP appears to be stronger; however, a definite quantification is not possible as the number of cells per cluster can vary. When cells had been cultured in the presence of 0.2 mM FC, hypointense regions corresponding to GFP or TfR1:GFP cell aggregates could be easily identified via MR (Fig. 5), and similar results were obtained in the presence of 2 mM FC (Supporting Information). From these results, it appears that, in the presence of FC supplementation, *TfR1* overexpression in CHO‐K1 cells does not lead to any noticeable enhancement in MR detection sensitivity in this model.

## 3 Discussion

To evaluate the effectiveness of *TfR1* as an MR reporter gene we have generated a CHO‐K1 cell line that expresses a bicistronic TfR1:GFP reporter. Overexpression of the *TfR1* transgene led to a decrease in endogenous *TfR1* mRNA levels. This was not unexpected because the 3′ untranslated region (UTR) of the endogenous *TfR1* mRNA contains five iron responsive elements (IREs), which ensures that the mRNA is degraded when intracellular iron concentrations are high 24, 25, 26. To prevent the *TfR1* transgene from being regulated by this negative feedback loop, we used the previously described strategy of deleting the IREs from the 3′UTR, which enables high levels of the exogenous *TfR1* mRNA to accumulate in the cells 11, 19. Consequently, we were able to achieve a more than 60‐fold increase in TfR1 protein levels in the CHO‐K1 cells.

Overexpression of TfR1 had no effect on *Fth1* mRNA levels, but resulted in a 13‐fold increase in Fth1 protein. It is well established that, in response to increasing intracellular iron concentrations, the binding activity of iron regulatory proteins to the IRE located within the 5′UTR of ferritin mRNAs is reduced, promoting protein translation 27. Therefore, the observed increase in Fth1 protein in the CHO‐K1 reporter line is most probably due to increased intracellular iron concentrations resulting from the overexpression of *TfR1* leading to its storage within ferritin, which is ultimately responsible for the generation of *T*_2_ contrast. However, a recent study from our group showed that overexpression of *TfR1* in mMSCs did not lead to an increase in Fth1 protein levels 17. This is probably because TfR1 protein increased by only 15‐fold in the mMSCs, compared with more than 60‐fold in the CHO‐K1 cells. It is thus likely that, in the mMSCs, the concentration of intracellular iron in the labile iron pool did not reach the threshold required to increase the rate of *Fth1* mRNA translation above baseline levels. Interestingly, a recent paper has shown that the concentration of intracellular iron required to trigger ferritin expression, and hence iron storage, can vary between different cell types 28, suggesting that for MRI detection some cell types may require higher levels of TfR1 protein expression than others.

In the absence of FC supplementation, overexpression of *TfR1* in CHO‐K1 cells led to a more than threefold increase in the concentration of intracellular iron. When cultured in serum‐containing medium, cells obtain iron from holo‐transferrin, which is a constituent of the FCS used in standard cell culture media 29. However, we have recently shown that, without FC supplementation, overexpression of the same *TfR1* transgene in mMSCs did not increase the concentration of intracellular iron, despite the fact that both cell types were cultured in the presence of 10% FCS. Likewise, an earlier study has shown that overexpression of *TfR1* in a mouse neural stem cell (mNSC) line did not significantly increase intracellular iron concentrations in the absence of FC supplementation 19. The ability of the CHO‐K1 cells expressing the TfR1:GFP^+^ reporter to accumulate iron in the absence of FC is probably due to the expression of very high levels of the recombinant TfR1 protein. These observations are important because they indicate that *TfR1* might not be an effective MR reporter for cell types that do not efficiently express recombinant proteins, for if the expression level of recombinant TfR1 protein cannot be raised above a certain threshold it may not be possible to increase the concentration of intracellular iron above background levels (at least under physiological conditions), rendering the cells undetectable with MRI.

In the presence of 0.2 mM FC, *TfR1* overexpression in CHO‐K1 cells led to an approximate twofold increase in intracellular iron. However, this did not lead to any noticeable changes in MR contrast (*T*_2_ relaxation), which was probably because the increase was very low and close to background levels. The increase in intracellular iron in *TfR1*‐expressing CHO‐K1 cells is in contrast to mMSCs, where under the same FC supplementation conditions overexpression of *TfR1* did not lead to any increase in intracellular iron levels 17. It is important to note that, for both cell types, supplementing the culture medium with FC increased intracellular iron concentrations to a greater extent than did overexpressing *TfR1*; for CHO‐K1 cells supplementation with 0.2 mM and 2 mM FC led to a respective fivefold and 10‐fold increase in intracellular iron, and for mMSCs to a respective 10‐fold and 40‐fold increase 17. This was also the case for mNSCs, where supplementation with 1 mM FC led to an approximate 10‐fold increase in intracellular iron 19. Taken together, these data indicate that, for all cell types tested to date, FC supplementation appears to be a much more effective means of increasing intracellular iron concentrations than does overexpressing *TfR1*, suggesting that to track cells with MRI it might be sufficient to simply incubate cells in the presence of FC shortly before implantation, rather than undertaking the more involved procedure of genetically manipulating the cells so that they overexpress *TfR1*. This is also the case for the ferritin reporters, where, similarly to *TfR1*, overexpression does not always increase intracellular iron concentrations as effectively as supplementation with FC 17, 30, 31, 32. However, such an approach would only be suitable for short‐term tracking, as it would be expected that with time the iron load would return to baseline levels and the cells would no longer be detectable via MRI. A further point to consider is that the concentration of intracellular iron appears to vary quite significantly depending on cell type, in both the presence and absence of FC (Table 1). For instance, the intracellular iron concentration of mNSCs 19 is about an order of magnitude lower than that observed with mMSCs 17, and when supplemented with FC two orders of magnitude lower than that of supplemented mouse dentritic cells (mDCs) 33. This suggests that some cell types might be easier to detect with MRI due to their intrinsically higher concentrations of intracellular iron or higher capacity to accumulate iron when supplemented with FC.

**Table 1:** Intracellular iron concentration of different cell types following FC supplementation

Cell type	Intracellular Fe without FC (pg/cell)	Concentration of FC* used in medium (mM)	Intracellular Fe with FC (pg/cell)	Fold increase	Ref.
hMSCs	0.08	0.25	0.34	4	39
CHO‐K1	0.01	0.20	0.05	5	this study
mNSCs	0.002	1.0	0.02	10	19
mMSCs	0.02	0.20	0.23	10	17
mB16F10	0.15	0.20	1.90	13	31
rGlioma F98	0.25	0.50	3.50	14	30
mDCs	0.06	0.25	2.15	36	33

When exact values were not mentioned in the studies these were estimated from the charts presented.

*Some studies employ ferric ammonium citrate, the *M*_w_ of which is slightly higher than that of FC, as a supplement.

Our *in vivo* imaging results showed that CHO‐K1 can be detected with MRI following injection into chick midbrain, even when the cells have not been supplemented with FC. This was probably related to the short relaxation time of the chick embryo brain, which leads us to suggest that relaxometric properties of a tissue might have an impact on the detection sensitivity of implanted cells. Our results contrast with previous studies involving the tracking of mNSCs in the mouse brain, where, in the absence of FC, cells were not detected *in vivo* with a 7 T MR scanner irrespective of the presence of reporter genes 19. This suggests that, in the absence of iron supplementation, the intrinsic amount of iron present in mNSC cells is not enough to produce contrast in the brain of mice, even when using a reporter gene.

Although our results and those of others 19 indicate that culturing cells in the presence of FC can generate sufficient contrast for MRI detection, this approach is unlikely to be as sensitive as labelling cells with SPIONs. With SPION labelling, intracellular iron concentrations of more than 5 pg/cell can easily be achieved 7, 34, and importantly the inherent superparamagnetism of the SPIONs means that, in contrast to FC, MR detection is not reliant on the availability of ferritin.

## 4 Conclusion

*TfR1* has potential use as an MR reporter gene, but importantly only if it is possible to achieve the high levels of expression required to significantly increase both the concentration of intracellular iron and the levels of ferritin protein. In this study, we have demonstrated the feasibility of using *TfR1* as an MR reporter in CHO‐K1 cells, but it should be noted that these cells can be very efficiently transduced and tend to express high levels of recombinant proteins. Before attempting to use *TfR1* as a reporter gene in other cell types, it would be important to consider (i) the ease with which the cells can be transduced, (ii) their ability to express high levels of recombinant proteins and (iii) the concentration of intracellular iron required to trigger ferritin expression. To track cells with MRI in the short term, simply loading cells with iron by supplementation with FC is likely to be an easier and more effective strategy than overexpressing *TfR1*.

## 5 Materials and Methods

### 5.1 Cell culture

Chinese hamster ovary (CHO) K1 cell line (CCL‐61^TM^, ATCC, Teddington, UK) was cultured in Dulbecco's modified Eagle's medium (DMEM) F12 containing 10% fetal calf serum (FCS) at 37 °C under a humidified atmosphere with 5% CO_2_. Culture plates/dishes were treated with 0.1% (w/v) gelatine solution at room temperature for at least 15 min, prior to seeding. All culture media and supplements were purchased from Sigma‐Aldrich, Gillingham, UK, unless stated otherwise.

### 5.2 Generation of lentiviral constructs and CHO‐K1 transduction

Mouse *TfR1* (NM_011638.4) cDNA was synthesized from an RNA sample of murine MSC D1 cell line (CRL‐12424™, ATCC, Teddington, UK). Iron responsive elements were excluded from amplification as previously described 17. *TfR1* cDNA was cloned into the pHIV‐eGFP plasmid (21373, Addgene, Cambridge, MA, USA). Viral production and titration methods were performed as previously described 17, 35. For CHO‐K1 transduction with lentiviral particles, cells (10^3^ cells/well in a 48‐well plate) were transduced with a multiplicity of infection (MOI) of 100 for 16 h in the presence of Polybrene (8 µg/mL). Transduction of cells was performed in three independent experiments (*n =* 3). After transduction, cells were allowed to expand for 7 days and then subcultured every 3–5 days. Non‐transduced cells were maintained at the same passage number.

### 5.3 Flow cytometry and fluorescence microscopy

Expression of GFP was evaluated via flow cytometry using a BD FACSCalibur instrument (BD Biosciences, Oxford, UK), with a 488 nm excitation laser and FL1 detector, and via fluorescence microscopy using a Leica DM IL inverted fluorescence microscope coupled to a Leica DFC420C camera.

### 5.4 RT‐qPCR and western blotting

For RT‐qPCR, 9 × 10^4^ cells were collected three passages after transduction, and cDNA synthesis and RT‐qPCR (CFX Connect™ real‐time PCR detection system, Bio‐Rad, Hemel Hempstead, UK) were performed according to the manufacturer's instructions (Fast SYBR® Green Cells‐to‐CT™ Kit, Life Technologies, Paisley, UK). The polyadenylate‐binding protein nuclear 1 (*Pabpn1*) and the vezatin adherens junctions transmembrane protein (*Vezt*) genes 36 were used as reference genes for data normalization. Primers used for all transcripts are detailed in the supporting information (Table S1). Data analysis was performed using the CFX system test software (Bio‐Rad), which applies the ΔΔCt method to normalize gene expression. For western blotting, 10^6^ cells were collected at passage 8 after transduction. All methodology and materials used for western blotting were as previously described 17. Anti‐ferritin heavy chain 1 (ab65080, Abcam, Cambridge, UK), anti‐transferrin receptor 1 (ab84036, Abcam) and anti‐actin (ab1801, Abcam) antibodies were used as primary antibodies and IRDye 680RD donkey anti‐rabbit (926‐68073, LI‐COR, Cambridge, UK) as a secondary antibody. Total protein gels were used for data normalization and actin was used as a reference to confirm data normalization.

### 5.5 Iron loading and quantification

To evaluate the concentrations of iron supplementation that CHO‐K1 cells tolerate, 5 × 10^3^ cells were seeded in 96‐well plates, cultured for 24 h, and then exposed to increasing concentrations of FC for 24 h. Viability was assessed using a Cell Counting Kit 8 (Sigma) according to the manufacturer's instructions. To measure intracellular iron loading, cells (5 × 10^4^ cells/well in a six‐well plate) were incubated for 4 days with ferric citrate (0.2 or 2 mM depending on the experimental conditions), and additionally 50 μM L‐ascorbic acid 37 and 1.28 mM human holo‐transferrin. Cells cultured in regular culture medium were used as controls. Intracellular iron was quantified by digesting 10^6^ cells in 100 μL 0.6 M HCl/0.14 M KMnO_4_ for 2 h at 60 °C. The digested cells were then reacted with 20 μL of ferrozine reagent (5 M ammonium acetate, 2 M ascorbic acid, 6.5 mM 3‐(2‐pyridyl)‐5,6‐diphenyl‐1,2,4‐triazine‐*p*,*p*′‐disulfonic acid monosodium salt hydrate and 15.4 mM neocuproine) for 30 min, leading to development of a coloured complex. The absorbance of the solution was read at 570 nm and iron was quantified using a reference curve obtained with an iron standard (TraceCERT™, Sigma) prepared under the same conditions.

### 5.6 Cell pellet relaxation measurements

Cells (10^7^) were cultured under the same conditions as described for iron uptake. Cells were then fixed with 4% formaldehyde, transferred to 0.2 mL polypropylene tubes and centrifuged for 30 min at 13 400 *g.* The supernatant was removed and 1% low‐melting agarose was used to cover the top of the cell pellet. Sagittal MR images were acquired with a Bruker 9.4 T Avance III HD instrument (Bruker, Coventry, UK) with a 40 mm transmit–receive quadrature volume coil using a multi slice multi echo (MSME) spin echo sequence with 25 echoes, an inter‐echo time of 8 ms and repetition time of 3577 ms. *T*_2_ relaxation maps were generated with ParaVision 6.0 (Bruker), from where the relaxation times of the cell pellets were obtained.

### 5.7 Fluorescence and MRI of chick embryos *in vivo*

For injection into chick embryo brains, 2 × 10^5^ cells, cultured under the same conditions as described for iron uptake, were suspended in saline containing deoxyribonuclease I (6 U/μL) and Fast Green (2 mM). Fast Green is used as a guide for the injection as it allows the monitoring of cell delivery at the site of the injection, but is quickly cleared from the embryo. Fertilized eggs of White Leghorn chicken were windowed at E3 as described previously 38 and the cells were then injected into the midbrain of embryos *in ovo* using a microcapillary pipette. After receiving the cells, embryos were allowed to grow for a further 48 h. Eggs were maintained at 38 °C and 40% humidity and all animal work followed UK regulations (consolidated version of ASPA 1986). At E5, embryos were removed from their eggs and imaged using a Leica M165FC stereomicroscope. The embryos were then fixed with 4% formaldehyde and mounted in 1.5 mL tubes containing 1% low‐melting agarose. Sagittal images of the chick embryos were acquired using a high‐resolution TurboRARE *T*_2_‐weighted sequence with the following parameters: field of view 35 × 15 mm^2^, matrix size 597 × 256, slice thickness 0.5 mm with no interslice gap, effective *TE* 39 ms, RARE factor 8, *TR* 2990 ms, averages 3, flip angle 90°, scan time 4 min 47 s.

## Supporting information

Supporting info item
