# Supplementary material for: Evaluating the effectiveness of transferrin receptor‐1 (TfR1) as a magnetic resonance reporter gene
Source: Contrast Media Mol Imaging. 2016 Feb 29;11(3):236–44. doi: 10.1002/cmmi.1686 (PMC4981909; doi:10.1002/cmmi.1686)
Supplement: Supplementary file 1 — Supporting info item [file CMMI-11-236-s001.docx]

**SUPPORTING INFORMATION**

**Evaluating the effectiveness of transferrin receptor-1 (TfR1) as a magnetic resonance reporter gene**

**Short title: Evaluating the effectiveness of TfR1 as a MR reporter gene**

**Sofia M Pereira ^1^, Anne Herrmann ^2^, Diana Moss ^1^, Harish Poptani ^1^, Violaine See ^2^, Steve R Williams ^3^, Patricia Murray ^1^, and Arthur Taylor ^1,*^**

^1^ Institute of Translational Medicine, University of Liverpool, Crown Street, Liverpool, United Kingdom; E-mails: [sofiamp@liverpool.ac.uk](mailto:sofiamp@liverpool.ac.uk) (S.M.P.); [dijim@liverpool.ac.uk](mailto:dijim@liverpool.ac.uk) (D.M.); harish.poptani@liverpool.ac.uk (H.P.); p.a.murray@liverpool.ac.uk (P.M.); [taylora@liverpool.ac.uk](mailto:taylora@liverpool.ac.uk) (A.T.)

^2^ Institute of Integrative Biology, University of Liverpool, Crown Street, Liverpool, United Kingdom; E-mails: [anne.herrmann@liverpool.ac.uk](mailto:anne.herrmann@liverpool.ac.uk) (A.H.); violaine@liverpool.ac.uk (V.S.)

^3^ Centre for Imaging Sciences, Oxford Road, University of Manchester, Manchester, UK, E-mail: [steve.williams@manchester.ac.uk](mailto:steve.williams@manchester.ac.uk)

***Corresponding Author:** taylora@liverpool.ac.uk; Tel.: +44-151 794 5450

**Table S1. Primer Pairs Used for RT-qPCR**

| **Primer** | **Origin** | **Forward Primer  (5’-3’)** | **Reverse Primer  (5’-3’)** | **Product (bp)** |
| --- | --- | --- | --- | --- |
| Transferrin Receptor-1 (*TfR1*) (exogenous) | *Mus musculus* | TGAGTGGCTACCTGGGCTAT | CTCCTCCGTTTCAGCCAGTT | 74 |
| Transferrin Receptor-1 (*TfR1*) (endogenous) | *Cricetulus griseus* | CAGGCAATTCAGAAATCATCCAAGA | CAGCTGCTTGATGGTGTCAGTG | 128 |
| Ferritin heavy chain-1 (*Fth1*)  (endogenous) | *Cricetulus griseus* | TGAGGAGAGGGAGCATGCCGA | CCAGTCATCACGGTCTGGTTT | 100 |

**
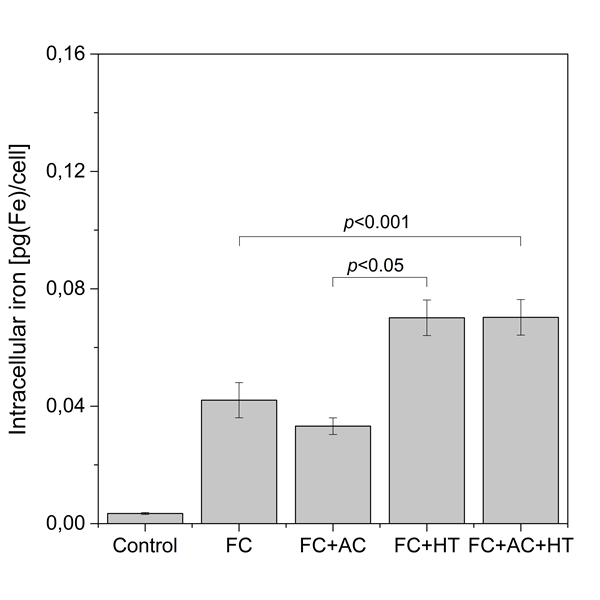
**

**Figure S2. The intracellular iron concentration of CHO K1 cells after exposure to 0.2 mM ferric citrate (FC) or a combination of 0.2 mM FC, 50 µM ascorbic acid (AC) and 1.28 mM human holo-transferrin (HT).**

**
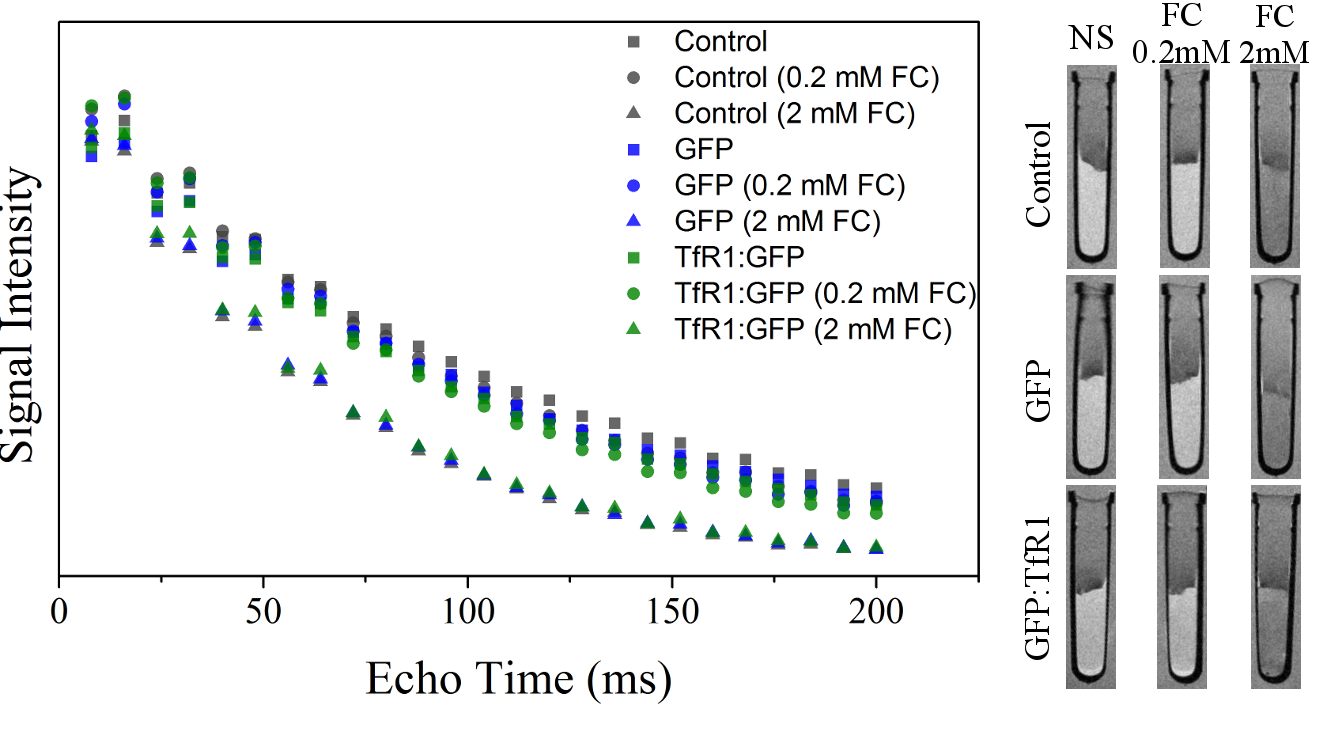
**

**Figure S3. The signal vs. TE from which T_2_ relaxation times have been derived. The right panel displays images of the cell pellets at a TE of 72 ms.** The signal intensity was obtained from a region of interest drawn in middle of the cell pellet.

**
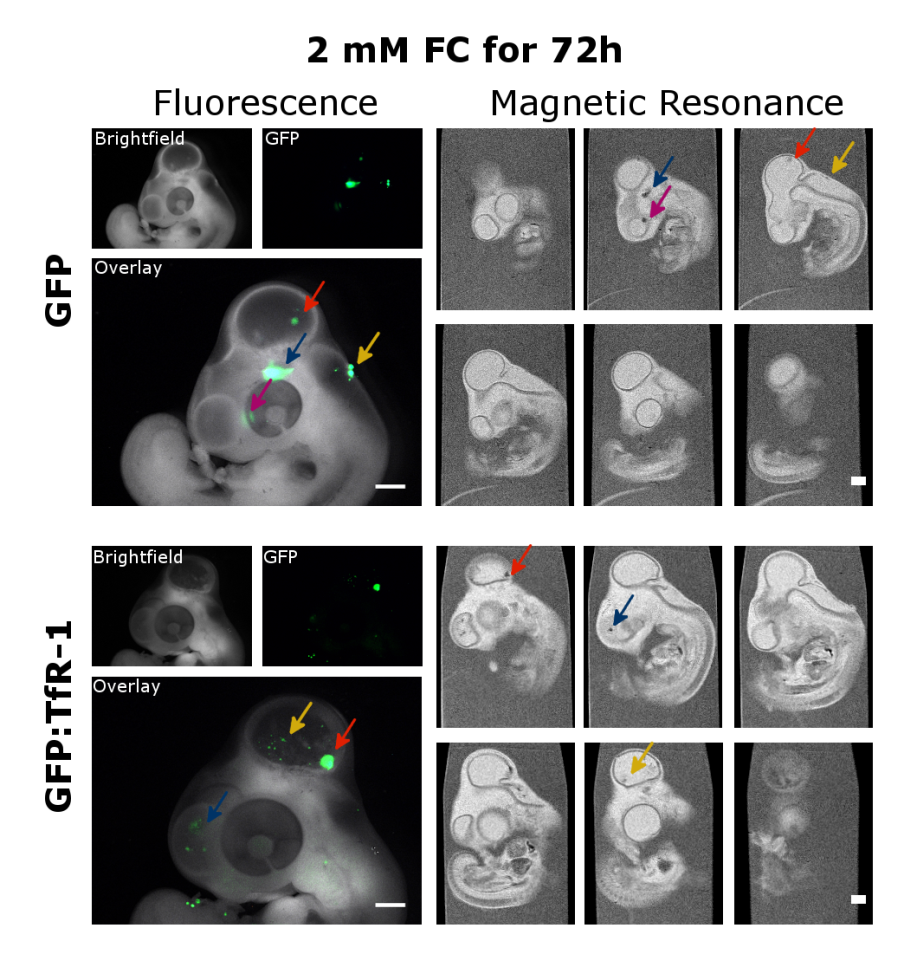
**

**Figure S4. Fluorescence and magnetic resonance imaging of cells implanted into the brain of a chick embryo.** Cells (2x10^4^) expressing GFP or TfR1:GFP were cultured with 2 mM FC for 72h and then implanted into the midbrain of chick embryos at embryonic day 3. At embryonic day 5 the embryos were harvested from their eggs, imaged with a fluorescence stereomicroscope and fixed prior to MR imaging using a T_2_-weighted RARE sequence. Clusters of cells expressing GFP were found in different regions of brain via fluorescence microscopy (left panel) and are indicated with arrows. Those clusters could then be identified as hypointense regions in the MR images. Six sagittal slices through the chick are shown in the right panel. Scale bars represent 1 mm.
